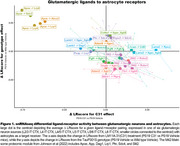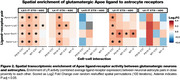# Integrated single‐nucleus and spatial analysis of PS19 tauopathy mice finds aberrant neuronal to glial communication is ameliorated by neurotrophin modulator LM11A‐31

**DOI:** 10.1002/alz70859_099554

**Published:** 2025-12-25

**Authors:** Robert R. Butler, Tao Yang, Crystal Han, Yann Le Guen, Kevin C Tran, Harry Liu, Song Albert Leng, Stephen M. Massa, Frank Longo

**Affiliations:** ^1^ Stanford University, Stanford, CA USA; ^2^ Quantitative Sciences Unit, Department of Medicine, Stanford University School of Medicine, Stanford, CA USA; ^3^ SFVAHCS & University of California San Francisco, San Francisco, CA USA; ^4^ Wu Tsai Neurosciences Institute, Stanford University, Stanford, CA USA

## Abstract

**Background:**

Tau pathology is a key driver of neurodegeneration in Alzheimer’s Disease (AD) and related dementia. Previous studies have shown that LM11A‐31 (C31)—a small molecule modulator of the p75 neurotrophin receptor (p75NTR)—reduced pathological tau accumulation, preserved synaptic plasticity, reduced degeneration of synaptic spines, and decreased microglial activation in tauopathy models. However, its effects on neuronal‐glial interactions remain unclear. Here, we investigate the impact of long‐term C31 treatment on synaptic‐glial communication using single‐nucleus RNA sequencing (snRNA‐seq) and spatial transcriptomics.

**Method:**

Tau^P301S^ (PS19) and wildtype (Wt) mice were dosed once daily by oral gavage with C31 or vehicle for 3 months from 6 months of age, when tau pathology was well established. Whole cortex was collected ∼1 hour after final dosing for 10x single‐nucleus RNA‐sequencing and CosMx spatial molecular imaging. Downstream analysis used Seurat and Giotto for cleaning and annotation, followed by manual curation of cell types. Cell‐to‐cell communication was assessed via Liana context factorization ligand‐receptor (LR) interaction analysis in snRNAseq and spatially enriched LR activity in CosMx.

**Result:**

Both astrocytes and microglia exhibited ligand‐receptor interactions with multiple glutamatergic neuronal subtypes in a genotype or drug‐dependent manner. Both glial types received glutamatergic neuronal Apoe signaling via glial lipoprotein receptors, including Lrp6, Lrp2, Abca1, Lrp1, Sorl1, and Lsr, in a spatially dependent manner. These interactions were elevated in PS19 mice but partially reversed with C31 treatment in select neuronal layers. Additionally, astrocytes as receivers showed a significant enrichment of extracellular matrix‐related signaling (Matrisome proteomic module from Johnson et al 2022; 7/32 members, p < 5.09e‐10), while as receivers microglia exhibited altered LR activity of p75NTR co‐receptor Sort1, and neurotrophin receptor Ntrk2.

**Conclusion:**

Our findings highlight the disruption of neuronal‐glial communication in tauopathy and suggest that C31 treatment ameliorates aspects of this crosstalk, potentially by directly engaging microglia in addition to its known protective role in synaptic integrity and plasticity. These results provide further insight into C31’s mechanism of action and its therapeutic potential for neurodegenerative diseases.